# Impairment of *Wnt11* function leads to kidney tubular abnormalities and secondary glomerular cystogenesis

**DOI:** 10.1186/s12861-016-0131-z

**Published:** 2016-08-31

**Authors:** Irina I. Nagy, Qi Xu, Florence Naillat, Nsrein Ali, Ilkka Miinalainen, Anatoly Samoylenko, Seppo J. Vainio

**Affiliations:** 1 Biocenter Oulu, Laboratory of Developmental Biology, Faculty of Biochemistry and Molecular Medicine, Oulu Center for Cell Matrix Research, University of Oulu, Aapistie 5A, Oulu, 90014 Finland; 2 InfoTech Oulu, University of Oulu, Oulu, Finland; 3 NordLab Oulu, Department of Clinical Chemistry, University of Oulu, Oulu, Finland

**Keywords:** Epithelial mesenchyme tissue interactions, Wnt signaling, Tubule morphogenesis, Glomerular cysts

## Abstract

**Background:**

Wnt11 is a member of the Wnt family of secreted signals controlling the early steps in ureteric bud (UB) branching. Due to the reported lethality of *Wnt11* knockout embryos *in utero*, its role in later mammalian kidney organogenesis remains open. The presence of Wnt11 in the emerging tubular system suggests that it may have certain roles later in the development of the epithelial ductal system.

**Results:**

The *Wnt11* knockout allele was backcrossed with the *C57Bl6* strain for several generations to address possible differences in penetrance of the kidney phenotypes. Strikingly, around one third of the null mice with this inbred background survived to the postnatal stages. Many of them also reached adulthood, but urine and plasma analyses pointed out to compromised kidney function. Consistent with these data the tubules of the *C57Bl6 Wnt11*^*−/−*^ mice appeared to be enlarged, and the optical projection tomography indicated changes in tubular convolution. Moreover, the *C57Bl6 Wnt11*^*−/−*^ mice developed secondary glomerular cysts not observed in the controls. The failure of *Wnt11* signaling reduced the expression of several genes implicated in kidney development, such as *Wnt9b*, *Six2*, *Foxd1* and *Hox10*. Also *Dvl2*, an important PCP pathway component, was downregulated by more than 90 % due to *Wnt11* deficiency in both the E16.5 and NB kidneys. Since all these genes take part in the control of UB, nephron and stromal progenitor cell differentiation, their disrupted expression may contribute to the observed anomalies in the kidney tubular system caused by *Wnt11* deficiency.

**Conclusions:**

The Wnt11 signal has roles at the later stages of kidney development, namely in coordinating the development of the tubular system. The *C57Bl6 Wnt11*^*−/−*^ mouse generated here provides a model for studying the mechanisms behind tubular anomalies and glomerular cyst formation.

**Electronic supplementary material:**

The online version of this article (doi:10.1186/s12861-016-0131-z) contains supplementary material, which is available to authorized users.

## Background

The Wnt signaling pathway plays important roles in several distinct processes during mammalian development including nephrogenesis [1]. Wnt11 is implicated in early kidney development since its absence leads to kidney hyperplasia due to reduced ureteric bud (UB) branching [2]. Whether Wnt11 has other roles in the embryonic or adult kidney remains unknown at present, but the fact that it is expressed in the UB tip of the maturing kidney is in line with the hypothesis of a possible role later in kidney development. It should be also noted that other members of the Wnt family have been implicated in kidney tubule development and tubular diseases leading to cystogenesis. The underlying mechanisms may stem from the roles of Wnts such as Wnt9b and Wnt7b in controlling the tubular luminal diameter and length via the planar cell polarity (PCP) pathway, since its deregulation is considered to be a cause of cystogenesis [3–5].

The glomerulocystic kidney (GCK) disease is one of human renal cystic disorders which could be both sporadic and inherited [6], and has a higher occurrence in infants than in adults [7]. Manifestation of this disease depends on the timing of exposure to the poorly characterized predisposing or causal factors influencing kidney development and function. Certain epigenetic modifiers may also contribute to the development of GCK disease [8] but the detailed molecular pathology behind remains poorly characterized. Although GCK disease may be caused by defects within the glomeruli, it is more typical for the cysts to develop as a secondary consequence to the anomalies in the tubules. Such anomalies can be connected not only with components of the Wnt signaling pathway, but also with HNF1β transcription factor, which is involved in polycystic kidney disease (PKD), and the polycystic complex proteins [3, 4, 9–11].

Because of the high mortality of *Wnt11* knockout mice (or *Wnt11*^*−/−*^) in a *SV129* background *in utero*, this model is not appropriate for studying the roles of Wnt11 at later stages in kidney development [2]. In the present study *SV129* mice with the *Wnt11* null allele were backcrossed with mice from the *C57Bl6* genetic background, which can differ notably from *SV129* mice in their anatomical features and physiological functions [12]. Analysis of these *C57Bl6 Wnt11*^*−/−*^ mice revealed that unlike the *Wnt11*^−/−^*SV129* mice some of them survived to adulthood, although they demonstrated prominent glomerular cysts and changes in kidney performance. The kidney tubules of the survivors were also enlarged and their convolution deviated from that of the controls. The *Wnt11*-dependent reduction of the expression of *Wnt9b*, *Six2* and certain stromal markers might point to a mechanism by which Wnt11 contributes to the development of the kidney tubular system. Thus *C57Bl6 Wnt11*^*−/−*^ mice may serve as a model for human glomerulocystic disease.

## Methods

### *C57Bl6 Wnt11*^*−/−*^ mice

The generation of the *Wnt11*^−/−^*129SV* mouse model has been previously described in [2]. The *Wnt11*^*−/−*^*SV129* mice for the present work were crossed with *C57Bl6* genetic background mice for a minimum of 10 generations. All the animal experimentation was authorized by the Finnish National Animal Experiment Board (ELLA) (62/2006) as being compliant with the EU guidelines for animal research and welfare.

### Histology, immunohistochemistry and electron microscopy

Kidneys were prepared from E16.5 embryos, newborn (NB) and adult mice (4–5 months old), fixed in 4 % paraformaldehyde and processed for tissue sections as described in [13]. Immunohistochemistry with the anti-Wnt11 antibody (Abcam) was performed using the tyramid signal amplification (TSA) kit (Perkin Elmer) as described in [13]. The Apoptosis TUNEL assays (Promega) were performed according to the manufacturer’s instruction as reported earlier [14]. Aquaporin-1 (AQP-1, Millipore), Aquaporin-2 (AQP-2, Sigma-Aldrich), thiazide-sensitive NaCl co-transporter (NCC, Millipore), acetylated α-tubulin (AT, Sigma-Aldrich), Phospho-Histone H3 (P-H3, Millipore) primary antibodies and Lotus Tetragonolobus Lectin (LTL, fluorescein labeled, Vector Laboratories), Dolichos Biflorus Agglutinin (DBA, rhodamine labeled, Vector Laboratories) lectins were used according to the manufacturers’ recommendations. Alexa Fluor 488 and 546-conjugated antibodies (Invitrogen) served as the secondary antibodies. DAPI (Sigma Pharmaceuticals) was used to stain the nuclei of the cells in the tissue sections. Electron microscopy samples were prepared as previously described [15] and examined using Phillips CM100 transmission electron microscope.

### Epithelial tubular cell and glomerular number

Epithelial tubular cells were quantified as previously described, with some modifications [4]. The numbers of epithelial cells per tubular cross-section were counted in 10 μm thick cryosections generated from the kidneys of the *Wnt11*^*−/−*^and control (WT) NB mice stained with DBA, LTL and DAPI. Those tubules that appeared to be round in shape were selected for the counting. 4–6 mice were used for making the sections, and nine slides were selected to represent key domains of the kidney (at least 100 μm between sections). Around 50 tubules per kidney were counted, representing groups to be analyzed when categorized by age (NB and adult) and genotype. For the number of glomeruli in WT and *Wnt11*^*−/−*^ (NB and adult), three mouse kidneys were sectioned and three sections were selected from each mouse for counting. Only glomeruli with intact shape and sectioned in the middle were recorded. The data were presented as the number of glomeruli per kidney section.

### RNA purification and quantitative RT-PCR

Total RNA was extracted from the kidneys of NB mice with the RNeasy mini kit (Qiagen). cDNA was synthesized from 1 μg of total RNA with the First Strand cDNA Synthesis Kit (Thermo Fisher Scientific). A sample from the cDNA library (2 μl at 1:10 dilution) in Brilliant II SYBR® Green QPCR Master Mix (Agilent Technologies) was subjected to qRT-PCR using the Mx3005P qRT-PCR System (Agilent Technologies) according to the manufacturer’s instructions. The primers for the qRT-PCR are described in Additional file 1: Table S1. GAPDH served as the reference for normalizing the qRT-PCR results. The PCR experiments were carried out in triplicates. Student’s *t*-test was used for the statistical analysis. Results were considered to be statistically significant when *p* < 0.05.

### Optical projection tomography

The three-dimensional organization of the kidneys was analyzed by optical projection tomography (OPT) according to Short et al. (2010) [16] at the developmental stages indicated in the results section. The ureteric tree of the kidney was stained with the anti-Troma-1 (Hybridoma Bank) antibody against cytokeratin, and detected with the Alexa Fluor 488-conjugated secondary antibody (Molecular Probes). A Bioptonics 3001 OPT scanner (Bioptonics, Edinburgh, UK) was used for image capture at a wavelength of 480 nm. The programs to analyze the OPT data and to generate the 3D images were ImageJ, Imaris 64 7.5.2 (Bitplane A.G.) and the filament-tracing tool of the Drishti V2.0.

### *In situ* hybridization

The protocol for the *in situ* hybridization was described by Wilkinson, 1992 [17]. The digoxigenin–labeled probes for *Wnt11* [13, 18], *Hox10* (a, c, d) and *Foxd1* [19], *Six2* and *Wnt9b* [20] were previously described.

### Biochemical tests using mouse plasma and urine

Four to five-months-old mice were placed in metabolic cages for 24 h, their urine and blood was collected, and biochemical assays (*n* = 8–10 animals/genotype) were performed in the Harwell Medical Research Council core laboratory (https://www.har.mrc.ac.uk/). Student’s *t*-test (*n* = 4–6) was used for the statistical analysis. Results were considered to be statistically significant when *p* < 0.05.

## Results

### Wnt11 is expressed in the epithelial tubular cells of the nephron and collecting duct during organogenesis

**Fig. 1:**
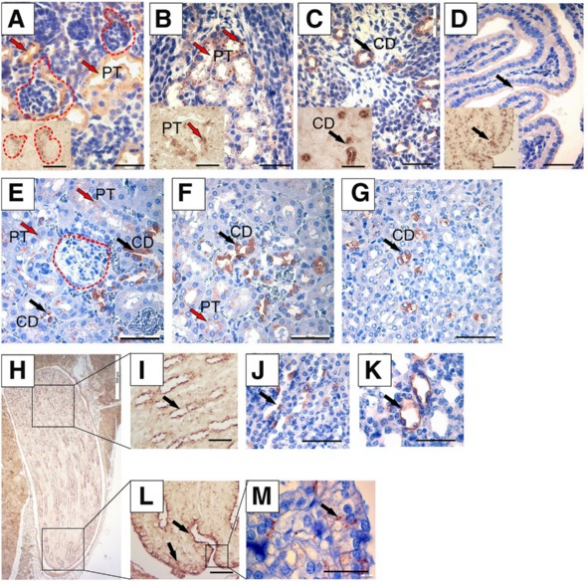
*Wnt11* is expressed in the cells of the cortical, medullary and papilla tubules, including the Bellini ducts of the terminal papilla. Kidneys from NB (**a**–**d**) and adult (**e**–**m**) mice were prepared and sectioned for *in situ* hybridization (**a**–**d** inserts, **h**, **i**, **l**) or immunohistochemistry (**a**–**d** large images, **e**–**g**, **j**, **k**, **m**). Counterstaining was performed with haematoxylin (**a**–**d** large images, **e**–**g**, **j**, **k**, **m**). **a**–**d** In the NB kidney, *Wnt11* is expressed in the maturing proximal tubules (PT) (**a**, red arrows), the more mature proximal tubules (**b** and insert, red arrows), the collecting duct (CD) tubules (**c** and insert, black arrows) and sporadically in the terminal papillary tubules (**d** and insert, black arrows). **e**–**g** *Wnt11*-positive cells in the cortical (**e**) and medullary (**f**, **g**) areas of the adult kidney. Expression is sporadic in the proximal tubule cells (**e**, **f**, red arrows) and more intense in the collecting duct cells (**e**–**g**, black arrows). **h**–**m**) The *Wnt11* gene and protein detected in the papilla of the adult mouse kidney is located in the papillary duct tubular cells (**i**, **j**, **k**, arrows) and Bellini ducts cells (**l**, **m**, arrows). *Wnt11* is not expressed in the glomerular tuft (**a**, **e**, areas circled by red dotted line). Bars: **a**–**g** and **i**–**m**, 50 μm; **h**, 500 μm

During the early stages of kidney development the *Wnt11* gene is expressed in the UB tip cells [2]. To ascertain whether Wnt11 might have putative roles later in development, we first examined whether it would be expressed in more advanced kidneys by *in situ* hybridization and immunohistochemistry. The investigations revealed that *Wnt11* is indeed expressed later, in the collecting duct (CD), the proximal tubule (PT), the terminal papillary duct cells, and the cells of the Bellini duct of the NB mice kidneys (Fig. 1a–d, arrows). Wnt11 is present in the adult mouse kidney in the CD (Fig. 1e–g, black arrows), the papillary ducts (Fig. 1h–m, arrows), and some cells of the PT (Fig. 1e, f, red arrows). No *Wnt11* expression was detected in the glomerular tuft cells (Fig. 1a, e, areas encircled by the dotted line). We conclude that *Wnt11* is expressed especially in the tubular system of the maturing kidney, which may suggest its later role in kidney development.

### *C57Bl6 Wnt11*^*−/−*^ mice can survive to adulthood

The *in utero* lethality of *Wnt11* knockout observed in the *SV129* genetic background prevented targeting of the putative later roles of Wnt11. In an effort to bypass this embryonic lethality, the null allele was transferred to the *C57Bl6* background mice by backcrossing *SV129 Wnt11*^*−/−*^ mice with the *C57Bl6* ones for a minimum of ten generations prior to analysis.

Strikingly, genotyping of the embryonic and NB progeny of the *C57Bl6 Wnt11*^*+/−*^ intercrosses revealed genotypes that were in accordance with the Mendelian ratio 50 % (+/−), 25 % (+/+) and 25 % (−/−) (*n* = 260). We conclude that the presence of the *Wnt11* null allele in the *C57Bl6* background partially overcome the *in utero* lethality reported in the *SV129* background [13].

However, the number of the surviving *Wnt11*^*−/−*^ mice had dropped by around 50 % (32/65, *n* = 65, *P* < 0.001) by the age of 4–6 weeks. An additional reduction of 10 % had taken place by the age of 12 months. The proportion of female to male *C57Bl6 Wnt11*^*−/−*^ survivors remain unchanged. Since the penetrance of the *Wnt11*^*−/−*^ is less marked in *C57Bl6* than in *SV129*, the generated *C57Bl6 Wnt11*^*−/−*^ mouse line could be a novel model for addressing the putative later roles of *Wnt11*.

### *Wnt11*^*−/−*^ influences kidney tubular system development in *C57Bl6* mice

**Fig. 2:**
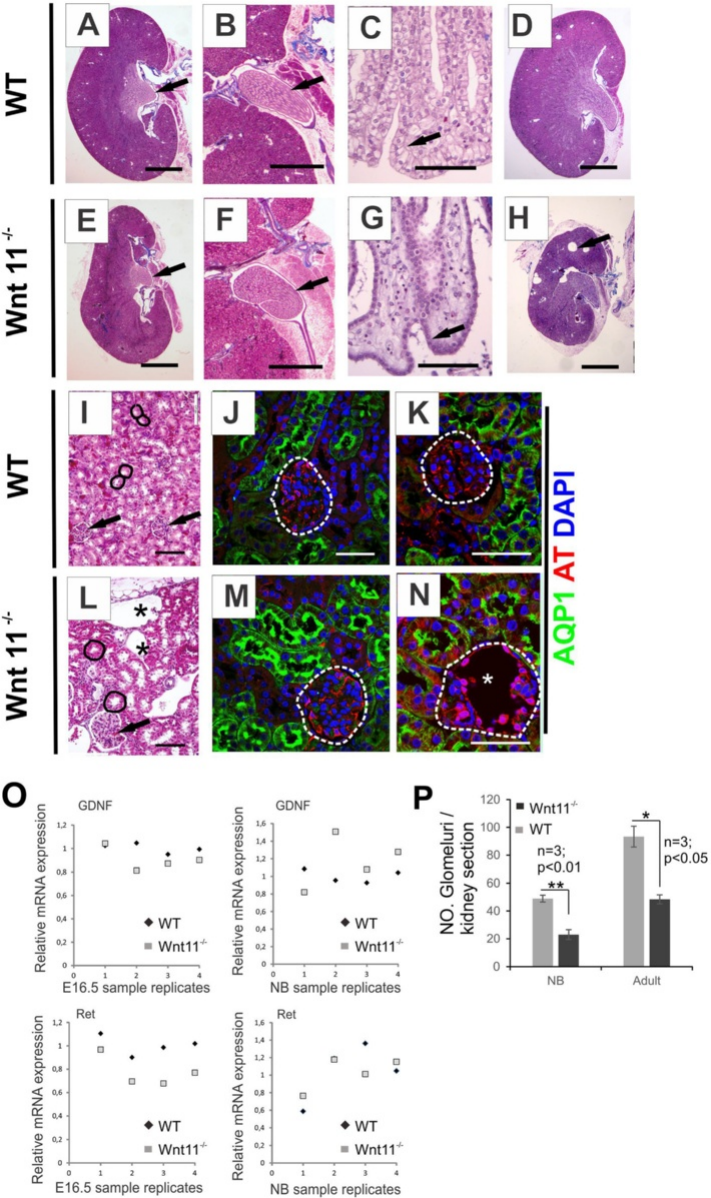
*Wnt11* ^*−/−*^ in *C57Bl6* mice leads to kidney hypoplasia, abnormal morphology of the papilla and Bellini ducts, and glomerular cysts formation. The kidneys were dissected from 4-5-months-old *C57Bl6 Wnt11* ^*−/−*^ and *Wnt11* ^*+/+*^ (WT) mice and sectioned rostro-caudally. **a**, **d** The WT kidney has a normal papilla morphology (arrow). **b** A higher magnification of WT papilla sections. **c** A high-power illustration of the epithelial cells in the region of the terminal papilla, showing Bellini duct cells with a columnar morphology (arrow). **e**–**g** *Wnt11*deficiency in the *C57Bl6* mouse leads to a hypoplastic kidney (compare **e** with **a**), with a typical abnormal appearance of the kidney papilla cells (compare **e**, **f** with **a**, **b**, arrows) and altered morphology of the Bellini duct cells towards a more squamous appearance (compare **g** with **c**, arrows). **h** An example of a very severe hypoplastic kidney in an adult *C57Bl6 Wnt11* ^*−/−*^ mouse with large cysts (arrow). **i**–**k** A WT kidney also depicting tubular cross-sections (**i**, circled areas) and glomeruli (**i**, arrow and **j**, **k**, dotted circled area). **l**–**n** Typical findings in *C57Bl6 Wnt11* ^*−/−*^ kidneys are cortical glomerular cysts in a rudimentary glomerular tuft (**l**, **n**, stars), enlarged proximal tubules (**l**, circled areas) and larger glomeruli (**l**, arrow). **m, n** Immunohistochemistry of proximal tubule marker Aquaporin 1 (AQP1) in green and glomerular podocytes cytoskeleton marker acetylated tubulin (AT) in red, nuclei marked with DAPI (blue). Note that the Wnt11 deficiency changes the Bowman capsule and the podocytes. No significant difference was noticed between the *Wnt11* ^*−/−*^ glomeruli and control glomeruli prior to cyst development (compare **m** with **j**, area circled by the dotted line). Note the intense AT staining in the glomerular rudiments of the *Wnt11* ^*−/−*^ kidneys (compare **n** with **k**, circled areas). **o** Relative mRNA levels of *GDNF* and *Ret* in *Wnt11* ^*−/−*^ and WT kidneys examined by qRT-PCR. The data were normalized to *GAPDH* expression. **p** Number of glomeruli per kidney section in *Wnt11* ^*−/−*^ and WT kidneys. **a**–**h**; **i**, **l** Masson Trichrome staining. Bars: **a**, **d**, **e**, **h** 2 mm; **b**, **f** 500 μm; **i**, **l** 100 μm; **c**, **g**, **j**, **k**, **m**, **n** 50 μm. *P < 0.05, **P < 0.01

Inspection of the *C57Bl6 Wnt11*^*−/−*^ mice after birth revealed that their kidneys were hypoplastic, as in the *SV129* model [13]. A panel of anomalies was found upon histological inspection of the kidneys. The most common anomalies were changes in the tubular system and the presence of small cysts in the kidney cortical region of NB and adult mice (Fig. 2a–h, i–n). The cysts were also present at E16.5 (Additional file 2: Figure S1, compare D with A). The number of glomeruli was significantly reduced both in NB and adult *Wnt11*^*−/−*^ kidneys as compared with the controls (Fig. 2p).

**Fig. 3:**
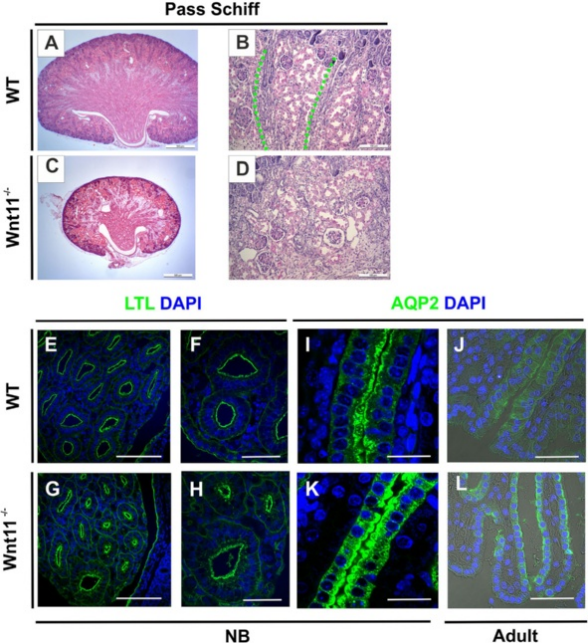
Wnt11 signaling is coupled to the development of the collecting duct and the kidney medullary tubular architecture. Adult WT and *Wnt11* ^−/−^ kidneys were prepared, sectioned and processed for immunohistochemistry with the collecting duct (CD) marker Aquaporin 2 (AQP2), the PT and loop of Henle marker lectin LTL, and DAPI, depicting the nucleus. Periodic Acid Schiff (PAS) staining was used for the histological examination. **a**–**d** The control kidney architecture, with longitudinal medullary rays consisting of the collecting ducts (green line), is deregulated in the *Wnt11* ^*−/−*^ mice (compare **c**, **d** with **a**, **b**). **e**–**l** Confocal microscopy images with phase contrast. Note the control morphology of the terminal papillary tubules and Bellini ducts (**e**, **f**) and the narrower cross-section of the Bellini ducts in the *Wnt11* ^−/−^ papilla (compare **g**, **h** with **e**, **f**). The high power micrographs depict AQP2 accumulation on the luminal side of the terminal papillary tubules in the WT (**i**, **j**), whereas AQP2 is also present on the basolateral side of the *Wnt11* ^*−/−*^ cells (**k**, **l**). The papilla epithelial cells at the tip region have adopted an appearance similar to squamous cells (compare **k**, **l** with **i**, **j**). **a**–**h**, **i**, **k**) NB. **j**, **l** Adult. Bars: **a**, **c** 500 μm; **b**, **d**, **e**, **g** 100 μm; **f**, **h**, **j**, **l** 50 μm; **i**, **k** 25 μm

**Fig. 4:**
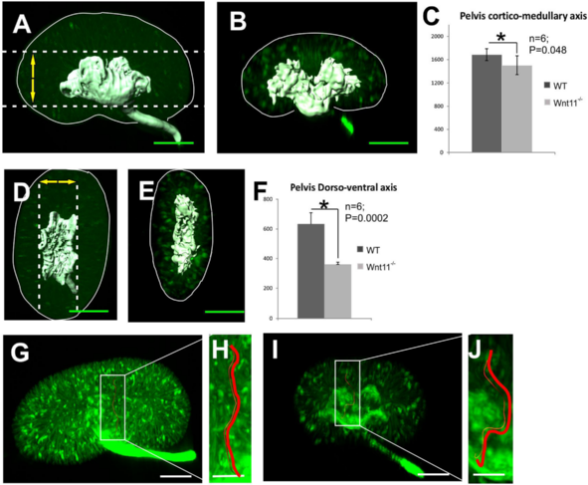
Optical projection tomography shows *Wnt11* dependent changes in the three-dimensional structure of the kidney. Kidneys of NB mice were dissected from the litters of *Wnt11* ^*+/−*^ mating’s, processed and stained as whole-mount specimens with cytokeratin-Troma-1 antibody, and then subjected to optical projection tomography (OPT). The OPT data were analysed with 3D Imaris and Drishti software to examine the influence of *Wnt11* knockout on the 3D layout of the kidney and its tubules. Troma-1 immunohistochemistry enables 3D presentation of the tubular projections in the kidney. Measurements of the *Wnt11* ^*−/−*^ and WT kidney pelvis axes in the cortical-medullary/rostro-caudal (**a**–**c**) and dorsal-ventral axis (**d**–**f**) views. *Wnt11* ^*−/−*^ kidney tubule reconstruction pointed to changes in the overall tubule convolution as compared with the controls. **g**–**j** One cortical-medullary tubule projection is highlighted in red in **j**, compare with (**h**). Bars: **a**, **b**, **d**, **e**, **g**, **i** 80 μm; **h**, **j** 300 μm

Closer inspection of the kidneys of the *C57Bl6 Wnt11*^*−/−*^ embryos showed disturbed tubules organization and abnormal convolution of the papilla as compared with the controls (Fig. 3, compare c, d with a, b, Fig. 4, g–j). The Bellini duct cells instead of being columnar in shape as it is normally the case, were more squamous in appearance due to the *Wnt11* deficiency (Fig. 2, compare g with c, arrows). Similar pattern was also shown with the Aquaporin 2 (AQP2) marker (Fig. 3, compare l with j). The lumina of the cortical tubules of the *C57Bl6 Wnt11*^*−/−*^ kidneys was enlarged relative to the controls, as observed in the hematoxylin and eosin staining (Fig. 2, compare l with i, circled areas). The proximal tubules (PT) and distal tubulues (DT) had an anomalous morphology, with a reduced tubular lumen and changes in cell polarity, as indicated by the LTL and AQP2 markers. This conclusion is based on the noted loss of polarized expression of AQP2 in the epithelial duct (Fig. 3, compare k with i).

We also studied by qRT-PCR the changes in expression of two major markers, *GDNF* and *Ret*, which were previously shown to cooperate with Wnt11 signals in regulating ureteric branching morphogenesis at early stages of embryo development in *SV129* mice [2]. We found that *Ret* expression was significantly downregulated at E16.5 in *C57Bl6 Wnt11*^−/−^ kidneys (*n* = 4, *p* ≤ 0.05), while *GDNF* was downregulated moderately (*n* = 4). In NB no significant differences in *Ret* and *GDNF* expression were observed (Fig. 2o). In summary, the *Wnt11* deficiency in the *C57Bl6* mice influenced the organization of the kidney epithelial tubular system.

### *Wnt11*-deficient kidneys are hypoplastic and glomerulocystic, with expanded tubules

Around 25 % (16/65) of the E16.5, NB and adult *Wnt11* deficient mice analyzed had severe kidney hypoplasia associated with the presence of relatively large glomerular and tubular cysts (Fig. 2d, h arrow, j, k, m, n, Additional file 3: Figure S2). The *Wnt11*^−/−^ tubular cysts were lacking primary cilia (Additional file 3: Figure S2I, J, arrows), while the non-cystic *Wnt11*^−/−^ tubules showed a normal appearance of the primary cilia (Additional file 3: Figure S2, compare f with b). Occasional collecting ducts (CD) cysts (Additional file 3: Figure S2H, arrows) could also be detected though it is a very rare phenomenon. In the *Wnt11*^*−/−*^ kidneys, the Bowman space was around 2–3 times larger than in the controls (Fig. 2, compare l, n, with i, k, star, Additional file 2: Figure S1, compare D, E, F, G with A, B, C, arrows) in line with the microcystic type characteristic for GCK disease [7]. To identify possible primary glomerular abnormalities that could lead to glomerular cyst formation we investigated noncystic glomerular structure by transmission electron microscopy (TEM). TEM showed no significant differences in precystic *Wnt11*^*−/−*^ glomeruli relative to the controls (Additional file 2: Figure S1, compare H with I).

The organization of the podocytes within the cystic glomeruli differed from the controls (Fig. 2, compare n with k, area encircled by the dotted line). The acetylated tubulin (AT) [21, 22] was also more prominent than in the controls (Fig. 2, compare m, n with j, k, in red). The glomeruli of the *Wnt11*^−/−^ kidneys had hypertrophied glomerular cysts as compared with the controls (Additional file 2: Figure S1C, F, G, black arrow), a finding that is in line with the reported resistance of *C57Bl6* mice to this condition [23, 24]. Cysts were noted to be distributed throughout the cortexes, indicating that both later and earlier assembled glomeruli can be affected by cystic formation. Interstitial fibrosis is detected in the severe kidney tubular anomalies of the *Wnt11*-deficient mice (Additional file 2: Figure S1F, empty arrow).

Given the *Wnt11* deficiency dependent changes in the kidney tubular system we also examined possible changes in the genes encoding the “core” PCP pathway and certain other genes (e.g. *TCS2*, *PDK1* and *HNF1b*) involved in cystic kidney diseases by qRT-PCR using E16.5 and NB kidneys. It has been shown that the expression of some of these genes such as *TCS2* was regulated by Wnt11 [25–27]. The qRT-PCR data revealed age-dependent and *Wnt11*^−/−^ dependent changes in the expression of several of the examined genes (Additional file 4: Figure S3). *Dvl2*, an important PCP pathway component, was downregulated by more than 90 % due to *Wnt11* deficiency in both the E16.5 and NB kidneys. Expression of the *Six2* and *Wnt9b* was reduced at E16.5, but not in the NB mice.

Taken together, our results indicate that the *Wnt11* deficiency in the *C57Bl6* mice disturbs the organization of the kidney tubular system and leads to glomerular cysts formation. The Wnt11 signal plays a role in the spatial arrangement of the tubular epithelial cells, partly by controlling the PCP pathway genes. However, since Wnt11 is not expressed in the glomerular cells (Fig. 1), the glomerular anomalies noted here are apparently secondary to the *Wnt11* knockout-dependent changes in the tubular cells of the nephron and collecting duct.

### *Wnt11* deficiency influences tubular convolution and the organization of the kidney pelvis

We next used optical projection tomography (OPT) technology to address the question if the *Wnt11* deficiency would influence any of three-dimensional parameters of the kidney. Analysis of the OPT data indicated a 34 % reduction in the volume of the *Wnt11*-deficient kidneys relative to that of the controls (376 ± 23 μm^3^ vs. 595 ± 81 μm^3^, *n* = 6-8, *p* < 0.05). Also the volume of the kidney pelvis (279 ± 11 μm^3^ vs. 418 ± 32 μm^3^, *n* = 6-8, *p* < 0.05) (Additional file 5: Figure S4A), its 3D appearance and relative size (Fig. 4a, b, d, e) were all changed when compared to control. The cortical-medullary axis was about 10 % smaller (1455 ± 115 μm vs. 1686 ± 101 μm, *n* = 6-8, *p* < 0.05) and the dorso-ventral axis was 56 % smaller (361 ± 15 μm vs. 634 ± 75 μm, *n* = 6-8, *p* < 0.05) in the absence of *Wnt11* function as compared with the controls (Fig. 4).

Computer-based reconstruction of the OPT images in the form of a 3D model also highlighted an increase in tubular convolution and length from the control values due to *Wnt11* deficiency (1751 ± 125 μm vs. 1520 ± 160 μm, *n* = 40, *p* < 0.05). One representative OPT-reconstructed tubule derived from the kidneys immunostained with the Troma-1 using whole mount technique (Additional file 5: Figure S4B-C´) is shown in Fig. 4g-j (in red). These results provide further support for the conclusion that Wnt11 signaling may be involved in some aspect of the organization of the kidney tubular system.

### Wnt11 signaling is involved in tubular organization

**Fig. 5:**
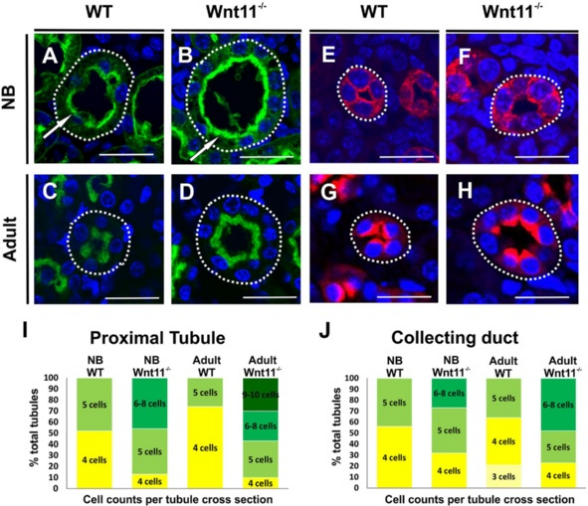
*Wnt11* deficiency leads to the heterogeneity in cell numbers in specific tubular segmental regions. Kidneys from WT and *Wnt11* ^−/−^ mice were prepared, sectioned and processed for immunohistochemistry with markers lectin LTL for the proximal tubule, in green, AQP2 for the collecting ducts, in red, and DAPI, for nuclei, in blue. **a**–**h** The staining of the cross-sections of the tubules at corresponding spatial location emphasizes that *Wnt11* deficiency leads to enlarged luminal diameter of the proximal tubules and collecting ducts in the kidney of NB (compare **b**, **f** with **a**, **e**, circled area) and adult mice (compare **d**, **h** with **c**, **g**, circled area). Note that the brush border, depicted with the LTL lectin marker, is more prominent and also abnormally organized in the proximal tubular cells of the *Wnt11* ^*−/−*^ kidneys relative to the controls (compare **b** with **a**, arrows). **i**, **j** Histograms showing estimated percentages of the tubules having the given numbers of cells per cross-section. The *Wnt11* deficient proximal and collecting ducts had increased percentages of tubules with an abnormally high cell number per cross-section relative to the values obtained from WT kidneys. Each colour indicates the number of cells/tubule cross-section (from 3 to 9–10 cells). Note that this phenotype was more obvious in the kidneys of the adult *Wnt11*-deficient mice. Bars: 25 μm

To substantiate the possibility that Wnt11 is involved in the control of the kidney tubular system we analyzed cross-sections of the PTs and CDs stained with AQP2 antibody and counterstained with DAPI and LTL. We counted the number of double positive cells per sections from NB and adult mice. The percentages of tubules with various numbers of cells per cross-section in the PT or CD of WT and *Wnt11*^*−/−*^ mice are illustrated in Fig. 5.

The histological inspection indicated that the size of the tubular lumen in the *Wnt11*^−/−^ kidneys was enlarged compared with the controls (Fig. 5a–h). Quantification of cell number in the tubular segments of the *Wnt11*-deficient kidneys showed notably higher variation in the total numbers of cells in both the PT and the medullary CD tubules (Fig. 5i, j). Around 40 % PTs of the NB *Wnt11*-deficient kidneys had 6–8 cells/cross-section, while the controls had only 4–5 cells/cross-section (Fig. 5i). This difference was even more apparent in the adults, where around 60 % of the PTs of the *Wnt11-*deficient kidneys had 6–8 or 9–10 cells/cross-section, while the corresponding values for the control sections were 4–5 cells/cross-section (Fig. 5i). Similar changes in the tubular cell count were found in the CD (Fig. 5j) in line with the roles of Wnt11 in cellular organization of the tubular epithelia.

### *Wnt11* influences tubular development by reducing cell proliferation, *Wnt9b* and *Six2* expression

**Fig. 6:**
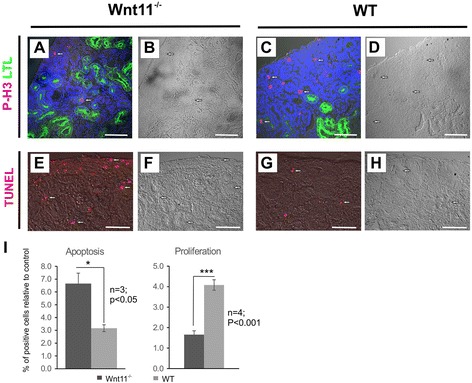
*Wnt11* deficiency reduces cell proliferation and increases apoptosis in the kidney. Kidneys were prepared from WT and *Wnt11* ^*−/−*^ mice and processed for immunohistochemistry. The sections were stained with phospho-Histone-3 (P-H3) antibodies, LTL and DAPI for cell proliferation and TUNEL for apoptosis. **a**–**d** Representative cell proliferation images obtained by confocal microscopy (**a**, **c**). Corresponding brightfield images are also shown (**b**, **d**). *Wnt11* ^−/−^ reduced cell proliferation in the nephron-forming cortex of *C57Bl6* mice, as judged by the presence of P-H3 staining (in red) mainly in the non-LTL^+^ cells (green) (compare **c**–**d** with **a**–**b**, several proliferating cells marked with arrows). **e**–**h** Cells in the developing cortex region underwent increased apoptosis in the absence of Wnt11 signaling, as indicated by the TUNEL assay (compare **g**–**h** with **e**–**f**, several apoptotic cells marked with arrows). **i** Quantification of TUNEL data (percentage of apoptotic cells in *Wnt11* ^−/−^ and control samples) and P-H3 data (percentage of proliferating cells in *Wnt11* ^−/−^ and control samples). Bars: 50 μm

Kidney hypoplasia and other changes in the tubular organization of *Wnt11*^−/−^ mice may involve changes in cell proliferation, apoptosis and also the expression of certain genes that are important for kidney development. To study these issues, cell proliferation was investigated by staining with the P-H3 antibody [28], while apoptosis was studied with the TUNEL assay (Fig. 6). These studies indicated that *Wnt11* knockout reduced cell proliferation, whereas cellular apoptosis was increased, especially in the kidney cortex, as compared with the controls (Fig. 6). These data are in line with the findings that kidney hypoplasia is caused by *Wnt11* deficiency (Figs. 2e, h and 3).

**Fig. 7:**
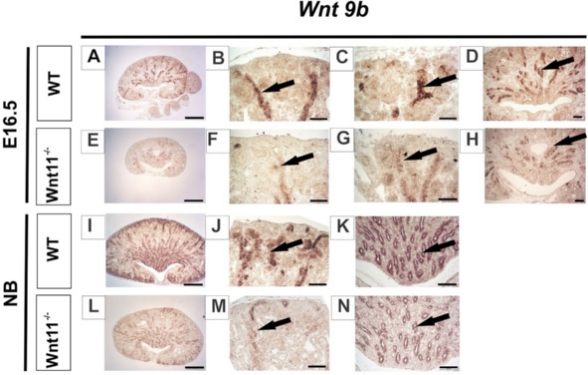
*Wnt9b*, a UB and nephron regulator signal, is reduced in *Wnt11* ^*−/−*^ kidneys. Kidneys were prepared from WT and *Wnt11* ^−/−^ mice, sectioned and subjected to *in situ* hybridization with the *Wnt9b* probe. **a**–**h** *Wnt9b,* which is expressed in the UB and the collecting duct (CD) cells derived from it, was notably reduced in the case of *Wnt11* knockout, especially in the CD cells situated in the subcortical region (compare **f**–**g** with **b**–**c**, arrows). Note the decreased expression of *Wnt9b* in the medullary CD as well (compare **h** with **d**). **i**–**n** *Wnt9b*remains reduced in the CD of the NB mouse kidney (compare **m**–**n** with **j**–**k**, arrows). Bars: **a**, **e**, **i**, **l** 500 μm; **b**, **c**, **f**, **g** 50 μm, **d**, **h**, **j**, **k**, **m**, **n** 100 μm

**Fig. 8:**
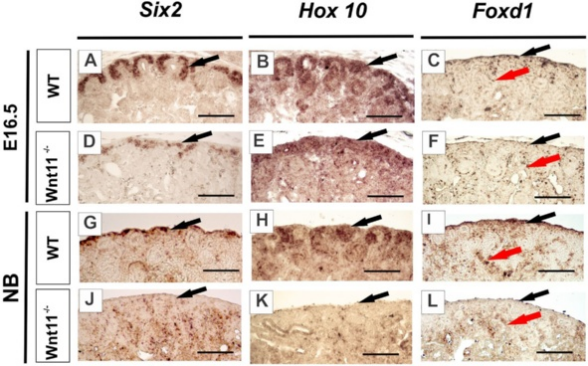
Failure in *Wnt11* function has an impact on the progenitors of the nephrons and the kidney stroma. Kidneys were prepared from WT and *Wnt11*-deficient mice, sectioned and subjected to *in situ* hybridization with the *Six2, Hox10* and *Foxd1* probes, depicting the nephron and the stromal progenitors, respectively. *Six2*, which is expressed in the nephron-forming progenitor cells, was severely reduced in the absence of Wnt11 (compare **d**, **j** with **a**, **g**, arrows). Similarly, *Hox10* and *Foxd1*, which are expressed in the precursors of the renal stroma, became deregulated at E16.5 and were reduced the neonatal mice (compare **b**, **c**, **h**, **i** with **e**, **f**, **k**, **l**, black arrows). Note that the *Foxd1* gene continues to be expressed in cells located deeper in the developing kidney (compare **f**, **l** with **c**, **i**, red arrows). The image exposure time was equivalent between all the samples of the same developmental stage for a given probe. Bar: **a**–**l**, 100 μm

In addition to the cellular changes in *C57Bl6 Wnt11*^*−/−*^ background mice, the expression of certain key genes that control kidney development was also deregulated. Expression of *Wnt9b* [4, 20, 29] was downregulated at E16.5 and NB as compared with the controls (Fig. 7, compare e–h, l–n with a–d, i–k, arrows), whereas *Wnt4* expression remained unchanged (data not shown). Likewise, the expression of *Six2*, a nephron progenitor marker gene, *Hox10* and *Foxd1*, stromal progenitor marker genes, was reduced in E16.5 kidneys of the *Wnt11*^*−/−*^ mice (Fig. 8a–l, arrows). In summary, changes in *Wnt9b, Six2, Hoxa10* and *Foxd1* expression may be involved in the development of the tubular dysmorphology in response to *Wnt11* deficiency in the kidney.

### *Wnt11* deficiency compromises kidney function

We were particularly interested in studying whether the changes noted in the kidney tubular system due to *Wnt11* deficiency would indeed be reflected by kidney functional performance in the adult mice. Creatinine clearance was reduced by around 52 % in the *Wnt11* deficient kidneys (*n* = 10, *p* < 0.05; Additional file 6: Figure S5A and Additional file 7: Table S2). Blood urea nitrogen (BUN) was higher relative to the concentration of plasma creatinine (*n* = 10, *p* < 0.05; Additional file 8: Table S3), whereas daily urine production was reduced in response to *Wnt11* knockout (Additional file 5: Figure S5C). Together these results indicate that *Wnt11* deficiency leads to a moderate degree of kidney failure and indicates a role for Wnt11 in the assembling of a functional kidney.

## Discussion

### Wnt11 signal coordinates kidney tubular system and lack of it promotes glomerular cyst development

The fact that some *C57Bl6 Wnt11*^*−/−*^ mice survive to adulthood provided a unique model for studying the roles of Wnt11 at later stages in the kidney development and maturation. *Wnt11* is expressed in kidney epithelial cells and its absence changed the morphology of the tubules by altering their diameter and convolution as observed by histochemistry and OPT. Unlike the *Wnt7b* and *Wnt9b* knockout models [3, 4], tubular cysts are not the predominant cystic phenotype in the *C57Bl6 Wnt11*^−/−^ model, but all the surviving mice had glomerular cysts and compromised glomerular function. Blood and urine analyses of *Wnt11*^−/−^ mice indicated moderate kidney failure.

Indeed, the *C57Bl6 Wnt11*^−/−^ kidney phenotypes show a definite correspondence to the characteristic of human GCK diseases [7, 8, 30, 31]. As within the *Wnt11* knockout, the *HNF1* gene mutation leads to hypoplastic GCK disease with an enlarged collecting duct lumen, which may point to synergistic roles for Wnt11 and HNF1 [30, 32, 33]. What could be the common ground for these phenotypes? Both *Wnt11* and *HNF1d* are expressed not only in the UB but also in the developing nephron, where they are functional. Moreover, since the UB initially induces the nephrogenesis program through Six2^+^ mesenchyme cells, the changes in the degree of UB bifurcation associated to its inductive signaling via Wnt9b, for example, may be reflected reciprocally in the number of eventually formed nephrons during kidney development. Since the Six2^+^ cell lineage, which was influenced by the *Wnt11* knockout also generates glomerular cells, cystogenesis could be promoted initially by Wnt11-dependent changes in the behavior of the nephron progenitor cells as well.

Our results obtained from the novel *C57Bl6 Wnt11*^−/−^ model and some other related ones [7, 8, 30, 31] raise the possibility that the compromised glomerular structure and function may occur secondarily to the primary changes that take place in the kidney tubules. In any case, the severity of these developmental anomalies in *Wnt11*^−/−^ kidneys depends to a dramatic extent on the genetic background of the mouse line. Our findings provide a valuable basis for identifying putative genetic modifiers that may eventually be relevant to the development of human cystic kidney disease [7, 8, 30, 31, 34]. Hence the *C57Bl6 Wnt11*^−/−^ mouse line reported here may serve as a novel Wnt model for addressing the detailed mechanisms of tubulogenesis and glomerular cyst development that are associated with human GCK diseases.

### Wnt11 is involved in the organization of kidney tubules through the planar cell polarity pathway

Apart from direct transcriptional control of target genes, Wnts are also known to modulate the PCP pathway that participate in various morphogenetic processes [35]. The *Wnt11* expression pattern found in the nephron and the collecting duct tubules correlates with that of *Wnt9b*, a PCP signal pathway involved in kidney tubulogenesis [4]. Like the kidneys of *Wnt9b*^−/−^ mice [4, 20, 29], the *C57Bl6 Wnt11*^*−/−*^ ones demonstrated an increased tubular diameter of the PT and the CD. Based on these data, we speculate that Wnt11 signaling may have a role in later nephron developmental stages being in part mediated via the Wnt9b.

*Wnt11* deficiency also changed the overall convolution of the tubules, as revealed by the OPT. Elongation and expansion of the tubules involve so-called “convergent extension” (CE) movements and oriented cell divisions. These are considered critical for the establishment of the tubular organization during kidney development [4]. It has been shown in other systems that Wnt11 signaling is connected with the control of CE movements as well [36–38].

### Wnt11 signaling may also take part in fine-tuning of nephrogenesis

The regulation of *Wnt9b* by *Wnt11* appears to be reciprocal, since *Wnt11* can be reduced by impairment of *Wnt9b* function in the kidney [4, 20, 28, 29, 39]. Indeed, the close relationships between Wnt11 and Wnt9b may provide a mechanism by which Wnt11 influences nephrogenesis. Expression of *Six2* is downregulated in the absence of either *Wnt9b* or *Wnt11*. How could *Wnt11* deficiency allow the maintenance of nephrogenesis, and simultaneously compromise the robustness of the process? We hypothesize that Wnt11 takes part in the fine-tuning of the Wnt9b concentration either by self-renewal of the Six2^+^ nephron progenitor cells or by induction of this population to proceed towards nephrogenesis in a well-coordinated manner [29, 40–43]. Thus, Wnt11 may fine-tune the output of Wnt9b signaling and in this way coordinate the self-renewal or commitment of the nephron progenitor cells in addition to controlling the *Six2* gene.

Wnt11 signaling may have a role in the differentiation of stromal progenitor cells, since the markers of this cell lineage, *Hox10* and *Foxd1*, were altered in the absence of Wnt11 function. Hox10 contributes to integration of the Foxd1^+^ progenitor into the kidney stroma in the developing kidney cortex but Foxd1 also has an impact on nephron progenitor cell differentiation [19, 44]. Hence Wnt11 may even have a broader role in coordinating the overall organization of the nephron and stromal progenitor cell layers during kidney development.

It should be noted that despite partial downregulation of *Wnt9b*, *Six2*, *Hox10* and *Foxd1* expression in *Wnt11*^−/−^ mice, the phenotype of *Wnt11*^−/−^ kidneys differs from the phenotypes described for the other knockout mice. Homozygous embryos for the *Wnt9b* allele die shortly after birth and have only rudimentary kidneys since *Wnt9b* transcription factor is essential for the development of mesonephric and metanephric tubules [20]. Also the inactivation of *Six2* gene results in premature differentiation of mesenchymal cells into epithelia and depletion of this progenitor cell population leads to severe renal hypoplasia [45]. On the other hand, conditional overexpression of *Wnt9b* in *Six2*-positive cells leads to formation of kidney tubular cysts and severe organ failure [46]. The observed defects in *Hox10* mutants include aberrant ureter branching, decreased nephrogenesis and a loss of kidney capsule formation [19]. The deletion of *Foxd1* causes striking developmental abnormalities such as pelvic fused kidneys likely due to the failure of the renal capsule formation [44]. Thus these genes are apparently only partially regulated by Wnt11 signaling.

One of the possible reasons for pathogenesis of cyst-like tubular structures in mice may by ureteral obstruction [47]. However, ureteral blockage in NB mice leads to gross enlargement of the kidney pelvis [48] while in our study the pelvis is smaller in *Wnt11*^*−/−*^ as compared to WT. On the other hand, the abnormal convolution of the kidney tubules with a decreased lumen size of the papillary tubules in *C57Bl6 Wnt11*^*−/−*^ kidney might impair the urine flow, which at the end could cause an increase in the upstream pressure in the glomerulus. We suggest that observed adult phenotype in our study results both from primary developmental kidney defects and chronic kidney diseases due to glomerular cysts, which has secondary impact on kidney development. However, in order to interpret the origin of the observed adult phenotype, various *Wnt11* conditional knockout models should be used in the future to specifically dissect Wnt11 functions during development and after birth, when kidney pelvis and tubules grow and mature.

## Conclusions

In summary, the novel *C57Bl6 Wnt11*^*−/−*^ mouse model introduced here revealed that the Wnt11 signaling pathway serves as an organizer of the UB, pretubular and stromal cells since the markers *Six2, Wnt9b,* and *Foxd1/Hox10* were reduced in its absence. Wnt11 also contributes to the spatial organization of the tubular epithelial cells in the assembling nephron in addition to taking part in the control of early UB branching. Dysfunction of Wnt11 signaling during tubule morphogenesis leads to the formation of glomerular cysts and secondarily to the impairment of glomerular function. These results point out that the *C57Bl6 Wnt11*^−/−^ mouse can be a useful model for gaining a better molecular understanding of the pathogenesis of human glomerulocystic kidney diseases [7, 30].

## Additional files

Additional file 1: Table S1. Primers used for qRT-PCR. (DOCX 16 kb) Additional file 2: Figure S1. Formation of glomerular cysts and anomalies of papillary duct due to *Wnt11* deficiency. A and D) Note discrete glomerular changes in E16.5 as the Bowman’s capsule of the glomerulus is enlarged and contains parietal podocytes (compare D to A, red arrows). B and E) Advanced cystic formation is found in NB, the glomerular tuft has an abnormal architecture when compared to the WT (arrow) and it is microcystic (star). C and F) Typical findings in all adult *Wnt11* ^*-/-*^ are cortical glomerular microcysts that contain rudiments of the tuft (F, G stars) and hypertrophied glomeruli (compare F to C, black arrows). In the severe kidney tubular anomalies of *Wnt11* deficient mice interstitial fibrosis is noted (F, empty arrow). G) High magnification of a cortical glomerular cyst (star) with dysmorphic tuft (arrow) in the kidney of *Wnt11* ^*-/-*^ mouse. H, I) TEM shows normal structure of non-cystic glomeruli in *Wnt11* ^*-/-*^ mice (RBC: red blood cell; US: urinary space; M: mesangial cell; GBM: glomerular basement membrane). J-M) Immunohistochemistry with AQP2 in kidney indicates that AQP2 staining is more intense in *Wnt11* deficient papillary ducts compared to controls (arrows). In contrast, the cell morphology of the cortical collecting duct was normal and expression of AQP2 is not different from controls (arrows). Bars: J, L 200 μm, A, D, C-G 100 μm, B, E, K, M 50 μm, H, I 2 μm. (JPG 1128 kb) Additional file 3: Figure S2. Cystic degeneration of kidney tubules without Wnt11 signaling. *Wnt11* knockout leads to a dilated proximal tubule (PT) with intense brush border lectin staining (compare E, I with A, arrows). 25 % of the *Wnt11* ^*-/-*^ mice have tubular cysts (compare G, H, I, J with C, D, arrows). Note the dilated Henle loop tubules in the medulla (compare G, J with C, arrows), the AQP1^+^ cyst (J, arrow) and CD AQP2^+^ cyst (H, arrows). *Wnt11* ^*-/-*^ tubular cysts were lacking primary cylia (I, J, arrow) while *Wnt11* ^*-/-*^ non-cystic tubules showed normal appearance of the primary cylia compared to control (compare F with B). Bars: A, B, E, F, I 50 μm; C, G, J 100 μm; D, H 50 μm. (JPG 666 kb) Additional file 4: Figure S3. qRT-PCR analysis of expression levels of “core” PCP signaling components and genes contributing to cystic kidney. The gene expression fold changes in *Wnt11* ^-/-^ kidney were normalized to the WT *Wnt11* ^+/+^ kidney for E16.5 and NB mice. The dotted line illustrates the expression levels in *Wnt11* ^+/+^ kidneys. The results from three independent experiments are shown. (JPG 2509 kb) Additional file 5: Figure S4. OPT-based kidney morphometric. *Wnt11* knockout (C, C´) alters the volumetric measures such as the relative volumes of the kidney and pelvis relative to WT (B, B´). (A) OPT volumetry data (*n* = 6-8). Drishti reconstruction of the pelvis (B, C) and highlighted regions depicted as B´ and C´ (boxed areas in B, C) revealing the sagittal/rostro-caudal tubular view. Note that the *Wnt11* deficiency led to considerable changes in the overall 3D arrangement of the tubules as well as the degree of their convolution when compared to control (compare C to B and C’ to B´). Bars: B, C 800 μm; B’, C’ 300 μm. (JPG 379 kb) Additional file 6: Figure S5.
*Wnt11* deficiency influences kidney function. Creatinine clearance (A) is significantly reduced in the *Wnt11* ^*-/-*^ mice when compared to WT, indicating reduced glomerular filtration rate (GFR). This situation corresponds to a diagnosis of mild to moderate renal failure in a human and is in line with the observed increase in blood urea nitrogen (BUN) (B) and reduced daily urine excretion (C). n = 8-10, *p* < 0.05. (JPG 146 kb) Additional file 7: Table S2. Urine biochemistry test in *Wnt11* ^*-/-*^ and WT mice. Creatinine clearance (ml/min) was calculated from the formula *UV/P* ×1/1440. *U*; urinary concentration of the substance (mmol/l)*, V*; urinary volume (ml), *P*; plasma creatinine concentration (μmol), 1440: minutes in 24 h. Creatinine clearance and urine volume were scaled to body weight. (DOCX 18 kb) Additional file 8: Table S3. Plasma biochemistry test in *Wnt11* ^*-/-*^ and WT mice. (DOCX 18 kb)

## Abbreviations

AQP-1: Aquaporin-1
AQP-2: Aquaporin-2
AT: Acetylated α-tubulin
CD: Collecting duct
CE: Convergent extension
DBA: Dolichos biflorus agglutinin
E16.5: Embryonic day 16.5
GCK: Glomerulocystic kidney
LTL: Lotus tetragonolobus lectin
NB: Newborn
NCC: Thiazide-sensitive NaCl co-transporter
OPT: Optical projection tomography
PCP: Planar cell polarity
P-H3: Phospho-histone H3
PKD: Polycystic kidney disease
PT: Proximal tubule
TEM: Transmission electron microscopy
TSA: Tyramid signal amplification
UB: Ureteric bud
Wnt11^−/−^: Wnt11 deficient
WT: Wild type
