# Supplementary material for: Impairment of Wnt11 function leads to kidney tubular abnormalities and secondary glomerular cystogenesis
Source: BMC Dev Biol. 2016 Aug 31;16(1):30. doi: 10.1186/s12861-016-0131-z (PMC5007805; doi:10.1186/s12861-016-0131-z)
Supplement: Additional file 1: Table S1. — Primers used for qRT-PCR. (DOCX 16 kb) [file 12861_2016_131_MOESM1_ESM.docx]

**Supplementary Table 1** Primers for qRT-PCR

|  | Forward primer | Reverse primer |
| --- | --- | --- |
| Vangle2 | CTAAGAGCCGGGATGGGAGTC | GGACGGACTTGGGCAGGTTG |
| Vangle1 | CACATCCGCTCTTTGCTTGCG | CACATCCGCTCTTTGCTTGCG |
| Dvl2 | CCATGAGTCTCGGACAGAACT | GTCCTCTCCATGCGTGGCGGC |
| Six2 | GTACGCTCACAACCCCTACC | TGAACCAGTTGCTGACTTGC |
| Wnt9b | TCCTGTGCTGTTCGTACCTG | GACAGCCGTGTCATAGCGTA |
| HNF1b | AGCCCACCAACAAGAAGATG | GGTCGTAGGCCTGGTACAAA |
| TCS2 | AAGACGAATCTGGCAGCCTA | CTGGTGTTTCCTGTGGGTCT |
| PDK1 | CTCACACTCTCTGGCCTCAC | AGCACACCAGGCTTTTAGCA |
| Nphp2 | CTGCGTACGGAGGGTACATC | CAGTGCAAAGCTGTTCGTCC |
| Nphp3 | GCAGAATGCTACTCCGTGGAT | TGACATAGAGGGCGTGTGTTG |
| Scrib | CCAGACTGAGGATGATGCCC | GTCTTCGAGACTTGGCAGGG |
| Prickle | CAGCTCCTCCTCAAGTTCGG | TGTTGTCGGGTATCCAGTGC |
| Wnt11 | GCCAAGTTTTCCGATGCTCC | CCTGTCTCCCCACTTCACTG |
| Wnt4 | GACAGCCGTGTCATAGCGTA | GGACGTCCACAAAGGACTGT |
| GDNF | TCCTGACCAGTTTGATGACG | CTGCCGCTTGTTTATCTGGT |
| Ret | GCTGCATGAGAATGACTGGA | CTGTTCCCAGGAACTGTGGT |
| GAPDH | AGAACATCATCCCTGCATCC | CAGTGAGCTTCCCGTTCAG |
