# Supplementary figures and images for: Impairment of Wnt11 function leads to kidney tubular abnormalities and secondary glomerular cystogenesis
Source: BMC Dev Biol. 2016 Aug 31;16(1):30. doi: 10.1186/s12861-016-0131-z (PMC5007805; doi:10.1186/s12861-016-0131-z)

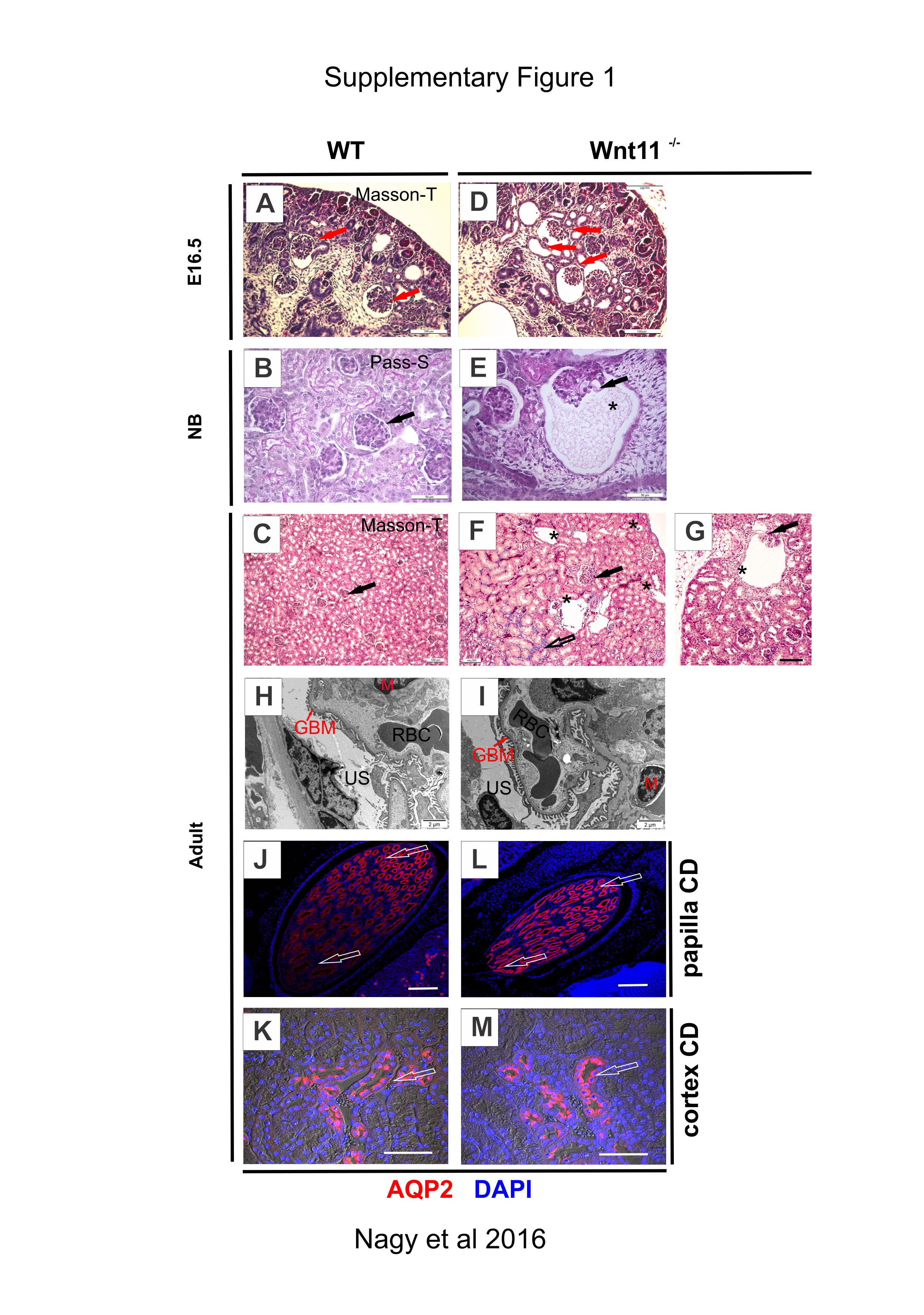

Supplement: Additional file 2: Figure S1. — Formation of glomerular cysts and anomalies of papillary duct due to Wnt11 deficiency. A and D) Note discrete glomerular changes in E16.5 as the Bowman’s capsule of the glomerulus is enlarged and contains parietal podocytes (compare D to A, red arrows). B and E) Advanced cystic formation is found in NB, the glomerular tuft has an abnormal architecture when compared to the WT (arrow) and it is microcystic (star). C and F) Typical findings in all adult Wnt11 -/- are cortical glomerular microcysts that contain rudiments of the tuft (F, G stars) and hypertrophied glomeruli (compare F to C, black arrows). In the severe kidney tubular anomalies of Wnt11 deficient mice interstitial fibrosis is noted (F, empty arrow). G) High magnification of a cortical glomerular cyst (star) with dysmorphic tuft (arrow) in the kidney of Wnt11 -/- mouse. H, I) TEM shows normal structure of non-cystic glomeruli in Wnt11 -/- mice (RBC: red blood cell; US: urinary space; M: mesangial cell; GBM: glomerular basement membrane). J-M) Immunohistochemistry with AQP2 in kidney indicates that AQP2 staining is more intense in Wnt11 deficient papillary ducts compared to controls (arrows). In contrast, the cell morphology of the cortical collecting duct was normal and expression of AQP2 is not different from controls (arrows). Bars: J, L 200 μm, A, D, C-G 100 μm, B, E, K, M 50 μm, H, I 2 μm. (JPG 1128 kb) [file 12861_2016_131_MOESM2_ESM.jpg]

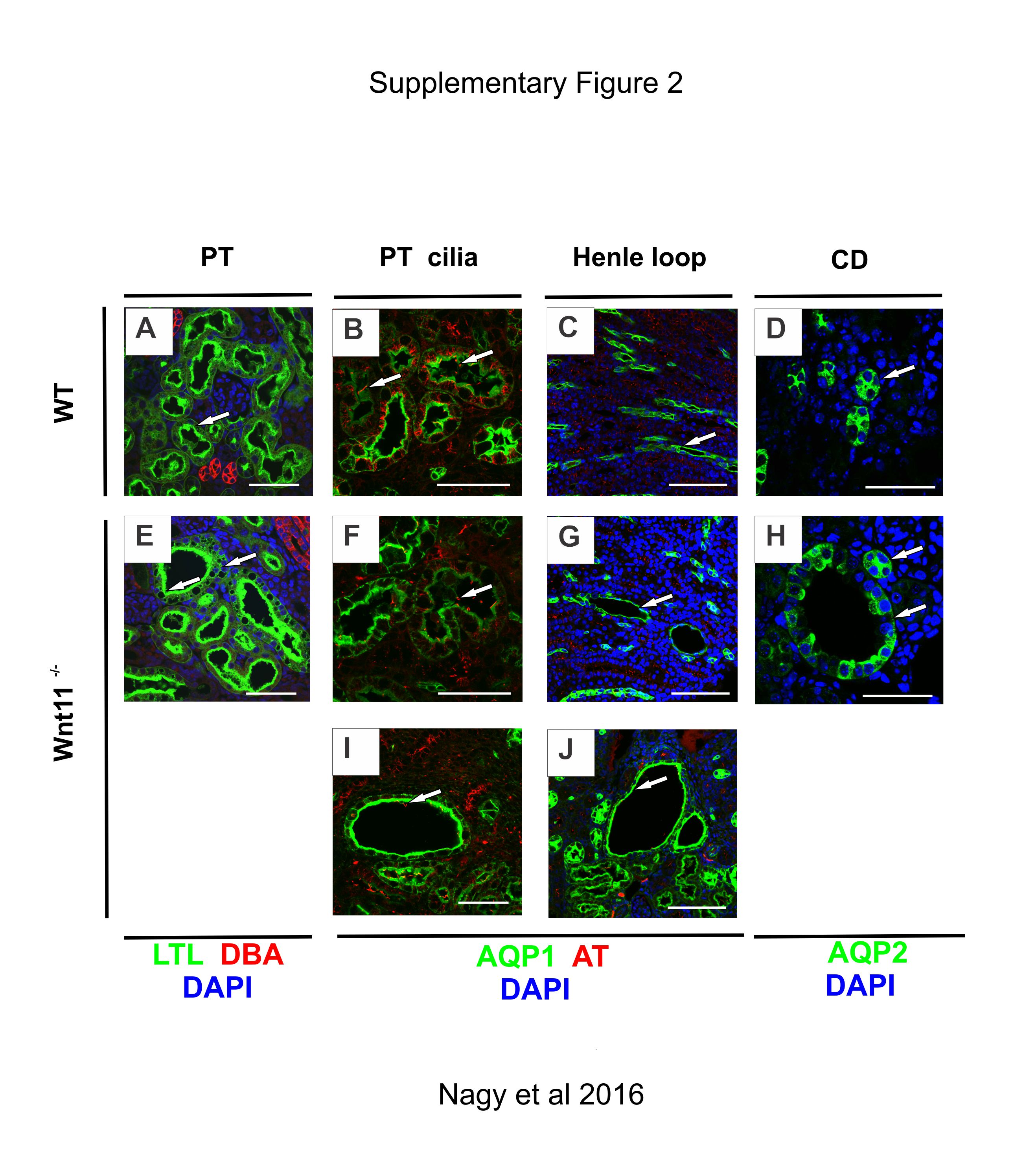

Supplement: Additional file 3: Figure S2. — Cystic degeneration of kidney tubules without Wnt11 signaling. Wnt11 knockout leads to a dilated proximal tubule (PT) with intense brush border lectin staining (compare E, I with A, arrows). 25 % of the Wnt11 -/- mice have tubular cysts (compare G, H, I, J with C, D, arrows). Note the dilated Henle loop tubules in the medulla (compare G, J with C, arrows), the AQP1+ cyst (J, arrow) and CD AQP2+ cyst (H, arrows). Wnt11 -/- tubular cysts were lacking primary cylia (I, J, arrow) while Wnt11 -/- non-cystic tubules showed normal appearance of the primary cylia compared to control (compare F with B). Bars: A, B, E, F, I 50 μm; C, G, J 100 μm; D, H 50 μm. (JPG 666 kb) [file 12861_2016_131_MOESM3_ESM.jpg]

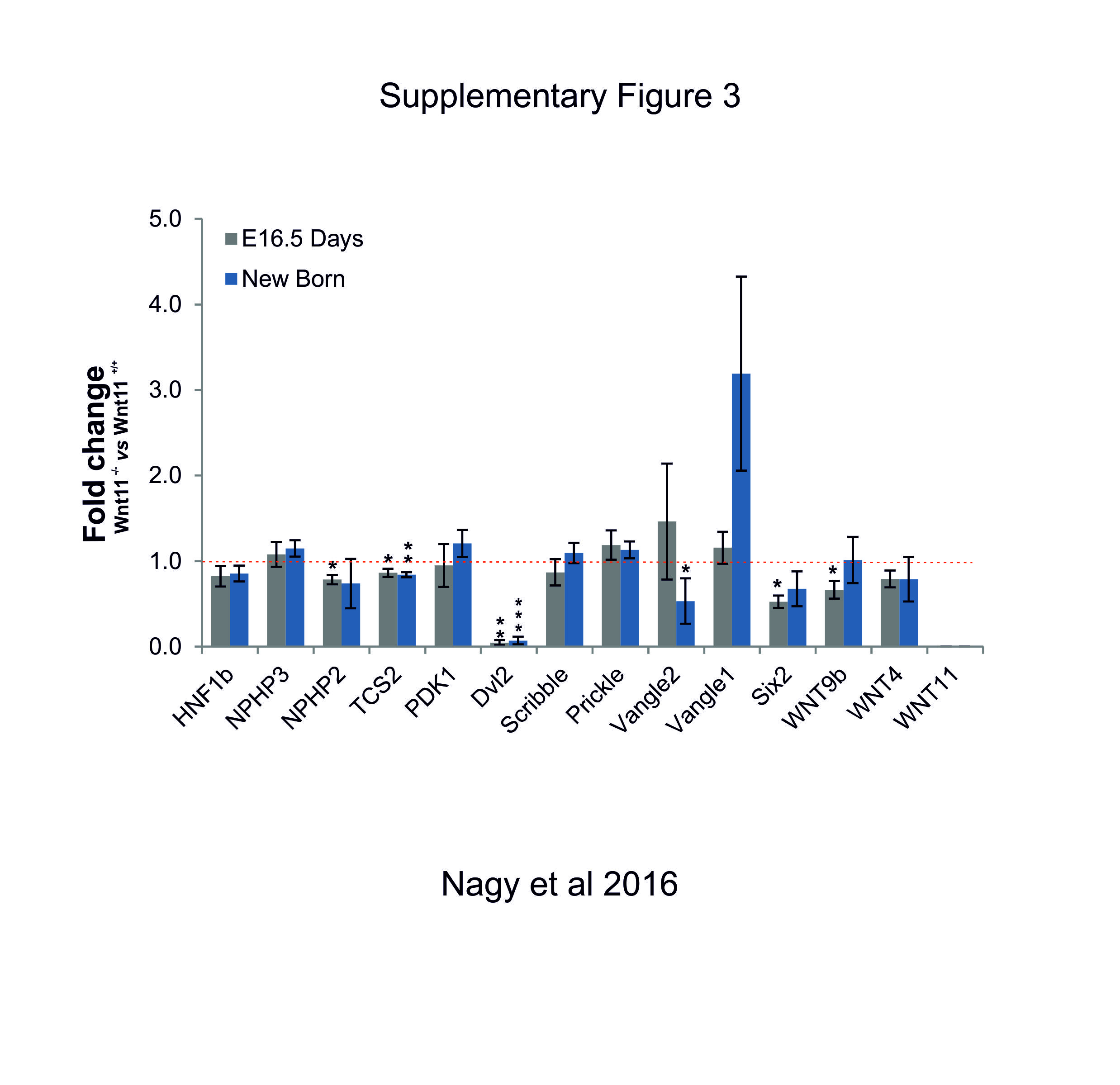

Supplement: Additional file 4: Figure S3. — qRT-PCR analysis of expression levels of “core” PCP signaling components and genes contributing to cystic kidney. The gene expression fold changes in Wnt11 -/- kidney were normalized to the WT Wnt11 +/+ kidney for E16.5 and NB mice. The dotted line illustrates the expression levels in Wnt11 +/+ kidneys. The results from three independent experiments are shown. (JPG 2509 kb) [file 12861_2016_131_MOESM4_ESM.jpg]

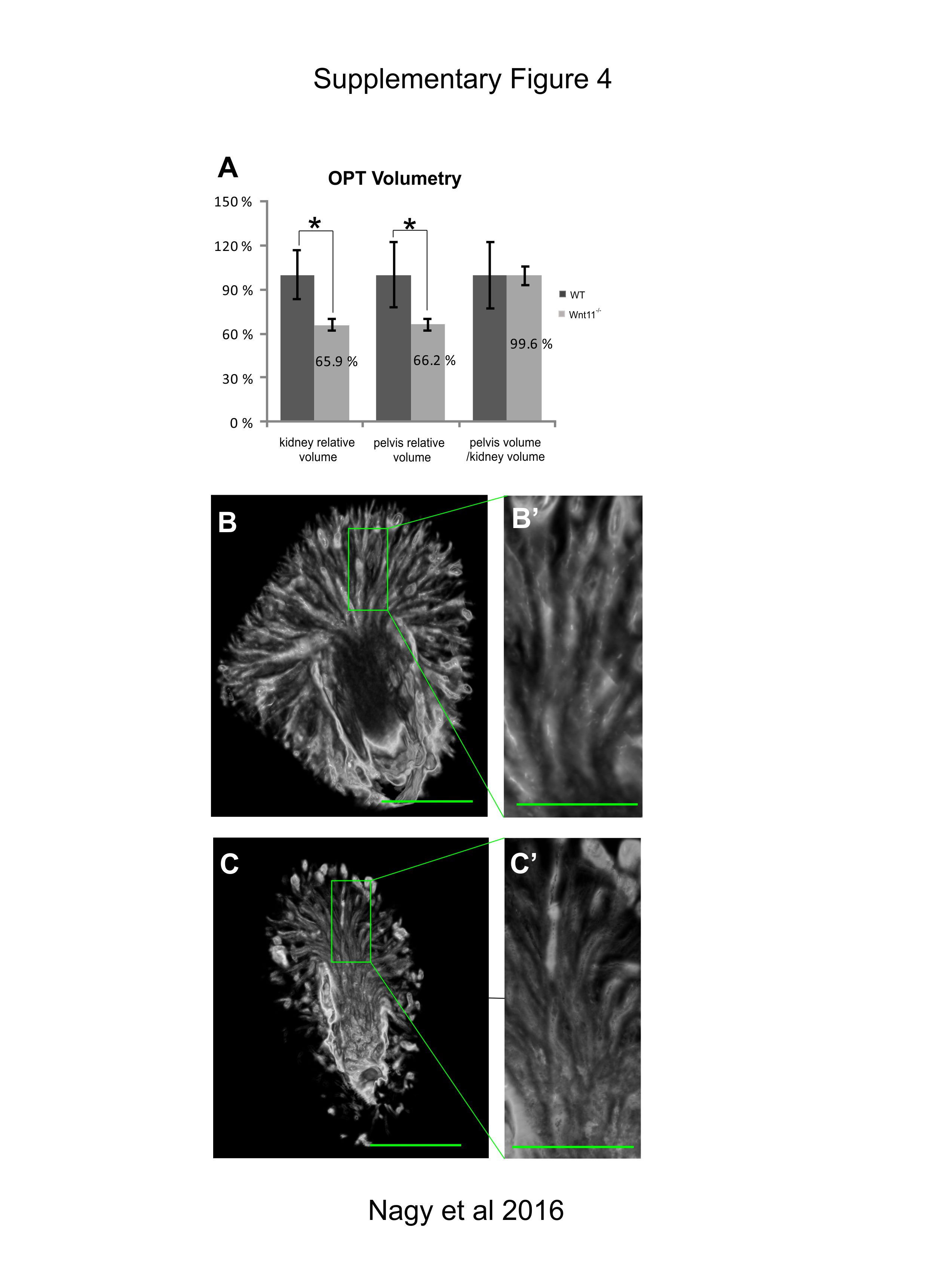

Supplement: Additional file 5: Figure S4. — OPT-based kidney morphometric. Wnt11 knockout (C, C´) alters the volumetric measures such as the relative volumes of the kidney and pelvis relative to WT (B, B´). (A) OPT volumetry data (n = 6-8). Drishti reconstruction of the pelvis (B, C) and highlighted regions depicted as B´ and C´ (boxed areas in B, C) revealing the sagittal/rostro-caudal tubular view. Note that the Wnt11 deficiency led to considerable changes in the overall 3D arrangement of the tubules as well as the degree of their convolution when compared to control (compare C to B and C’ to B´). Bars: B, C 800 μm; B’, C’ 300 μm. (JPG 379 kb) [file 12861_2016_131_MOESM5_ESM.jpg]

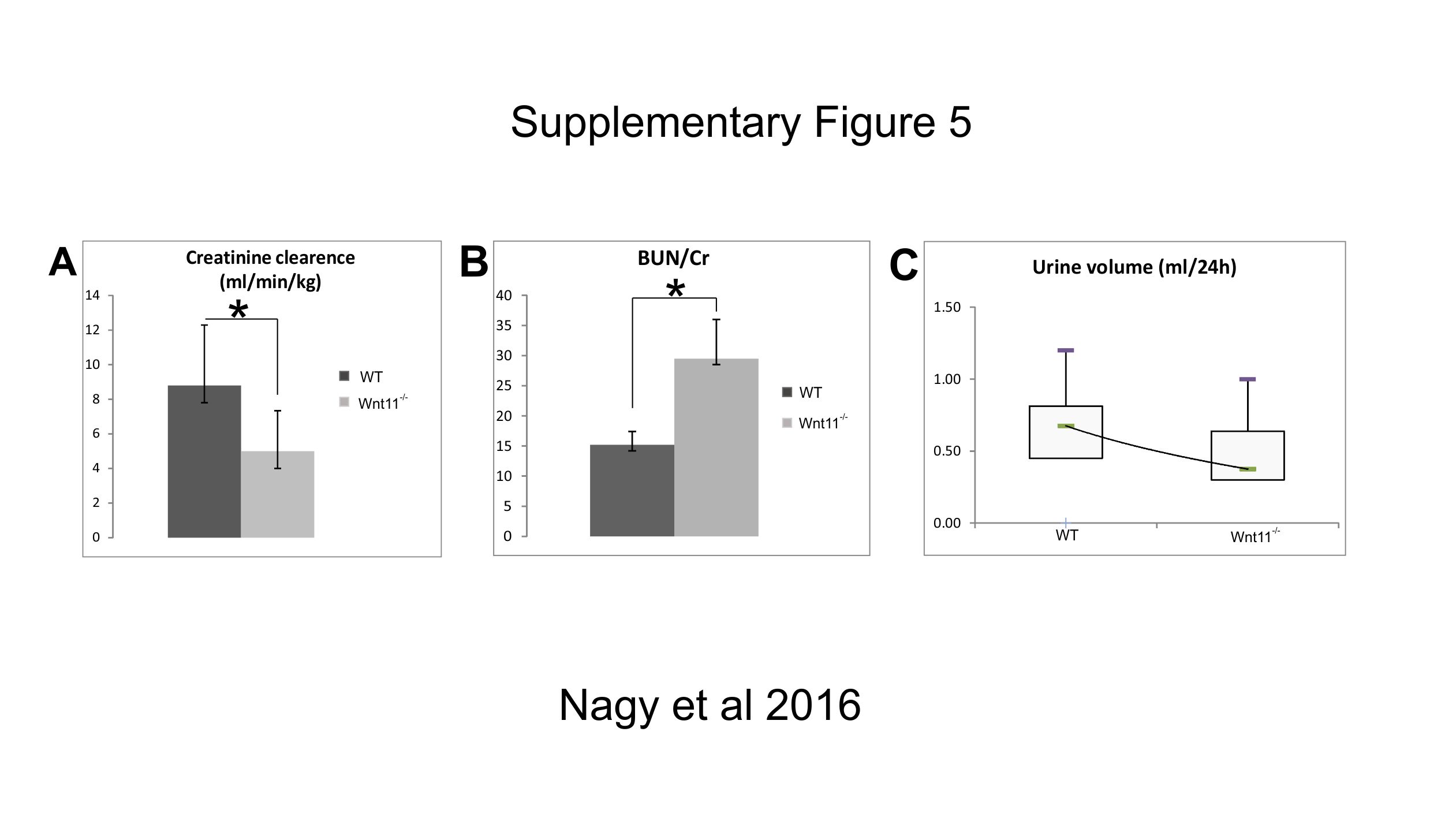

Supplement: Additional file 6: Figure S5. — Wnt11 deficiency influences kidney function. Creatinine clearance (A) is significantly reduced in the Wnt11 -/- mice when compared to WT, indicating reduced glomerular filtration rate (GFR). This situation corresponds to a diagnosis of mild to moderate renal failure in a human and is in line with the observed increase in blood urea nitrogen (BUN) (B) and reduced daily urine excretion (C). n = 8-10, p < 0.05. (JPG 146 kb) [file 12861_2016_131_MOESM6_ESM.jpg]
