# Supplementary material for: Impairment of Wnt11 function leads to kidney tubular abnormalities and secondary glomerular cystogenesis
Source: BMC Dev Biol. 2016 Aug 31;16(1):30. doi: 10.1186/s12861-016-0131-z (PMC5007805; doi:10.1186/s12861-016-0131-z)
Supplement: Additional file 7: Table S2. — Urine biochemistry test in Wnt11 -/- and WT mice. Creatinine clearance (ml/min) was calculated from the formula UV/P ×1/1440. U; urinary concentration of the substance (mmol/l), V; urinary volume (ml), P; plasma creatinine concentration (μmol), 1440: minutes in 24 h. Creatinine clearance and urine volume were scaled to body weight. (DOCX 18 kb) [file 12861_2016_131_MOESM7_ESM.docx]

| **Supplementary table 2** | |  | |  | |  |
| --- | --- | --- | --- | --- | --- | --- |
| Mean (SD) urine biochemistry obtained on day 3 of metabolic cage studies | | | | | | |
| Analyte | Unit | | WT (n=15) | |  | *Wnt11 ^-/-^*(n=15) |
|  |  |  |  |  |  |  |
|  |  | |  | |  |  |
| Na :Cr † | mmol/l | | 27,3 (12,8) | |  | 33,3 (13,7)n |
|  |  | |  | |  |  |
| K: Cr † | mmol/l | | 41,26 (20,3) | |  | 43,8 (22,1)n |
|  |  | |  | |  |  |
| Cl : Cr† | mmol/l | | 31,5 (21,4) | |  | 45,7 (22,3) n |
|  |  | |  | |  |  |
| Ca : Cr† | mmol/l | | 0,68 (0,2) | |  | 0,58 (0,14) n |
|  |  | |  | |  |  |
| PO4:Cr† | mmol/l | | 16,2 (4) | |  | 19,4 (8,8) n |
|  |  | |  | |  |  |
| ureea:Cr† | mmol/l | | 256 (122) | |  | 316 (126) n |
|  |  | |  | |  |  |
| osmolarity †† | mol/l | | 1450 (152) | |  | 1230 (202)n |
|  |  | |  | |  |  |
| 24 h protein creat | g/mmol | | 0,9 (0,6) | |  | 0,7 (0,5)n |
|  |  | |  | |  |  |
| Creatinine clearence ¶ | ml/min/kg | | 8,75 (3,5) | |  | 4,59 (2,3)* |
|  |  | |  | |  |  |
|  |  | |  | |  |  |
| † urinary sodium,potassium,calcium, phosphate, or ureea : creatinine ratio | | | | | | |
| †† mOSmol/l | |  | |  | |  |
| ¶ ml/min/kg | |  | |  | |  |
| n : no statistical significance when compared to WT group | | | | | | |
| * statistical significance P < 0,05 when compared to WT data set | | | | | | |
