# Supplementary material for: Impairment of Wnt11 function leads to kidney tubular abnormalities and secondary glomerular cystogenesis
Source: BMC Dev Biol. 2016 Aug 31;16(1):30. doi: 10.1186/s12861-016-0131-z (PMC5007805; doi:10.1186/s12861-016-0131-z)
Supplement: Additional file 8: Table S3. — Plasma biochemistry test in Wnt11 -/- and WT mice. (DOCX 18 kb) [file 12861_2016_131_MOESM8_ESM.docx]

| **Supplementary table 3** | |  | |  | | |  |
| --- | --- | --- | --- | --- | --- | --- | --- |
| Mean (SD)plasma biochemistry obtained on day 3 of metabolic cage studies | | | | | | | |
| Analyte | Unit | | WT (n=15) | |  | *Wnt11 ^-/-^*(n=15) | |
|  |  |  |  |  |  |  |  |
|  |  | |  | |  |  | |
| Na † | mmol/l | | 149 (1,3) | |  | 152 (2)n | |
|  |  | |  | |  |  | |
| K † | mmol/l | | 8,4 (0,8) | |  | 7,5 (1,2))n | |
|  |  | |  | |  |  | |
| Cl † | mmol/l | | 111,3 (2,4) | |  | 114,4 (3,3) n | |
|  |  | |  | |  |  | |
| Ca † | mmol/l | | 2,8 (0,1) | |  | 2,8 (0,06)n | |
|  |  | |  | |  |  | |
| PO4† | mmol/l | | 3 (0,2) | |  | 2,7 (0,3) n | |
|  |  | |  | |  |  | |
| Albumin †† | mmol/l | | 31,1 (1,3) | |  | 30,3 (0,5) n | |
|  |  | |  | |  |  | |
| Glucose | mol/l | | 19,2 (2,8) | |  | 17,4 (3,3) n | |
|  |  | |  | |  |  | |
| Creatinine¶ | g/mmol | | 17 (2,15) | |  | 19 (2,1) * | |
|  |  | |  | |  |  | |
| BUN | ml/min/kg | | 3 (0,5) | |  | 6,3 (1,4)** | |
|  |  | |  | |  |  | |
| BUN/Cr |  | | 15 (2,2) | |  | 30 (6,5) ** | |
|  |  | |  | |  |  | |
|  |  | |  | |  |  | |
| n : no statistical significance when compared to WT group | | | | | | | |
| * statistical significance P < 0,01 when compared to WT | | | | | | | |
| ** statistical significance P < 0,05 when compared to WT | | | | | | | |
|  | | | | | | | |
